# Nuclear Expression of KLF6 Tumor Suppressor Factor Is Highly Associated with Overexpression of ERBB2 Oncoprotein in Ductal Breast Carcinomas

**DOI:** 10.1371/journal.pone.0008929

**Published:** 2010-01-28

**Authors:** Ricardo C. Gehrau, Diego S. D'Astolfo, Catherine I. Dumur, José L. Bocco, Nicolás P. Koritschoner

**Affiliations:** 1 Centro de Investigaciones en Bioquímica Clínica e Inmunología (CIBICI-CONICET), Departamento de Bioquímica Clínica, Facultad de Ciencias Químicas, Universidad Nacional de Córdoba, Córdoba, Argentina; 2 Centro de Investigaciones en Química Biológica de Córdoba (CIQUIBIC-CONICET), Facultad de Ciencias Químicas, Universidad Nacional de Córdoba, Córdoba, Argentina; 3 Department of Pathology, Virginia Commonwealth University, Richmond, Virginia, United States of America; Health Canada, Canada

## Abstract

**Background:**

Krüppel-like factor 6 (KLF6) is an evolutionarily conserved and ubiquitously expressed protein that belongs to the mammalian Sp1/KLF family of transcriptional regulators. Though KLF6 is a transcription factor and harbors a nuclear localization signal it is not systematically located in the nucleus but it was detected in the cytoplasm of several tissues and cell lines. Hence, it is still not fully settled whether the tumor suppressor function of KLF6 is directly associated with its ability to regulate target genes.

**Methodology/Principal Findings:**

In this study we analyzed KLF6 expression and sub-cellular distribution by immunohistochemistry in several normal and tumor tissues in a microarray format representing fifteen human organs. Results indicate that while both nuclear and cytoplasmic distribution of KLF6 is detected in normal breast tissues, breast carcinomas express KLF6 mainly detected in the cytoplasm. Expression of KLF6 was further analyzed in breast cancer tissues overexpressing ERBB2 oncoprotein, which is associated with poor disease prognosis and patient's survival. The analysis of 48 ductal carcinomas revealed a significant population expressing KLF6 predominantly in the nuclear compartment (X^2^ *p* = 0.005; Fisher *p* = 0.003). Moreover, this expression pattern correlates directly with early stage and small ductal breast tumors and linked to metastatic events in lymph nodes.

**Conclusions/Significance:**

Data are consistent with a preferential localization of KLF6 in the nuclear compartment of early stage and small HER2-ERBB2 overexpressing ductal breast tumor cells, also presenting lymph node metastatic events. Thus, KLF6 tumor suppressor could represent a new molecular marker candidate for tumor prognosis and/or a potential target for therapy strategies.

## Introduction

Krüppel-like factor 6 (KLF6) belongs to a large family of mammalian Sp1/KLF transcription factors that play critical roles in regulating key cellular functions ranging from differentiation to proliferation and apoptosis [1], [2], [3], [4]. KLF6 is an evolutionarily conserved and ubiquitously expressed protein that was identified as an activator of pregnancy-specific genes [5], [6], [7]. Several independent reports proposed that KLF6 is a tumor suppressor gene product due to downregulation of KLF6 expression or frequent somatic inactivating mutations were found within the *klf6* gene in prostate carcinoma, colorectal tumors, glioblastoma, hepatocellular carcinoma and lung-derived tumors [8], [9], [10], [11], [12], [13], [14], [15]. Nevertheless, a number of other studies established that genetic alterations of KLF6 were infrequently observed in distinct types of human cancers or, in addition the *klf6* gene expression was enhanced in some tumors [8], [16], [17], [18], [19], [20], [21].

More recently, it has been reported that target disruption of the *klf6* gene caused early embryonic lethality and severely impaired embryonic stem cells proliferation [22]. In addition, p21^CIP1/WAF^, a transcriptional target gene for KLF6 in differentiated cells [12], [23], was not downregulated in *klf6* ^−/−^ embryos [22]. Most importantly, in hepatoma cell lines the situation is particularly interesting since KLF6 knockdown led to dephosphorylation of Rb together with downregulation of Cyclin D1, which in turn strongly impaired cell proliferation involving G1-S arrest [24]. Thus, the phenotypes described in *klf6*−/− mice, stem cells and hepatoma cell lines suggests that endogenous KLF6 is required for cell cycle progression, thereby contrasting with its potential tumor-suppressor activity. In agreement with these observations, knock down of KLF6 expression mediated by siRNA lead to reduction in cell cycle progression of hepatocarcinoma cell lines and they were more susceptible to DNA-damage induced apoptosis [25].

On the other hand, it has been reported that KLF6 has the ability to reduce cell proliferation rate through increased c-Jun degradation by the proteasome dependent pathway upon tumor promoter and proliferating cell signaling generated by phorbol 12-myristate 13-acetate and ionomycin [26]. More recently, phorbol 12-myristate 13-acetate-induced growth arrest of non-small lung cancer cells was mediated by induction of KLF6 expression following PKC activation [27]. In addition, KLF6 mediates inhibition of Cyclin D1/cdk4 activity, resulting in growth inhibition [28]. Re-expression of wild type KLF6 into prostate carcinoma PC-3 cells and non-small lung cancer cells, which harbor loss-of-function mutations or downregulation of the endogenous *klf6* gene, respectively, enhanced growth suppression and apoptosis [29], [15].

Current available information clearly demonstrates the involvement of KLF6 in the regulation of cell proliferation and apoptosis which can influence tumor development. However, cell context and/or biochemical signaling, interaction with specific transcriptional partners and/or sub-cellular distribution of KLF6 could manage the outcome of KLF6 function to different and opposite tumor pathways. Thus, differential regulation of KLF6 under specific cell environment provides a plausible explanation on the divergent information published so far about the tumor suppressor function of KLF6. In this regard, Guo and co-workers demonstrated a decreased binding of KLF6 to its DNA binding sequence on the Tissue Factor Pathway Inhibitor-2 (TFPI-2) gene due to CpG hypermethylation in highly invasive breast cancer cell lines, suggesting a tumor suppressor function of KLF6 [30]. However, siRNA mediated knockdown of endogenous KLF6 in the MCF7 breast cancer-derived cell line lead to reduced cell proliferation [25].

Breast cancer is the most common malignant, clinically heterogeneous disease in women, and metastatic events occurrence at distant sites originated at the primary tumor is the main cause of death. The characterization of the tumor aggressiveness of patients with breast cancer reside in the study of both, clinical and pathological parameters established by medical criteria such as tumor size, axillaries lymph node status, histological grade and angio-invasion. However, these clinical and pathological parameters seem to be not accurate enough for disease prognosis and treatment follow-up. A large number of proteins have been reported as potential molecular prognostic markers although few of them such as uPA/PAI1, steroid-receptor expression and *HER-2/neu* gene amplification and protein expression have been established at clinical level (reviewed in [31], [32], [33], [34], [35], [36]).

Within the Sp1/KLF family of transcription factors, only KLF4 and KLF5 have been further investigated in the context of breast tumor environment. The transcript and protein levels of KLF4 are increased during breast tumor progression and its nuclear localization has been associated to an aggressive phenotype in early stages of breast tumors [37], [38]. In opposite, the transcript of KLF5 is decreased in various breast cancer cell lines mainly as a consequence of chromosomal deletion or degradation of the KLF5 protein by the proteasome dependent pathway [39], [40], [41]. However, high expression of KLF5 in breast cancer samples was directly correlated with cell proliferation *in vivo* and with HER-2 expression level whereas patients had a shorter disease-free survival and overall survival time than those with lower KLF5 expression [42].

As an essential step to gain knowledge about function of KLF6 in breast tumors, the expression pattern and sub-cellular distribution of KLF6 protein was analyzed in samples of human breast cancer along with the expression of ERBB2 as a tumor aggressiveness marker. Interestingly, KLF6 was extensively detected in the nucleus of HER-2/ERBB2-overexpressing breast tumor cells whereas it was mainly cytoplasmic in the normal tissue counterpart. Moreover, this KLF6 expression pattern tends to be associated with small size and stage I ductal breast tumors, as well as with axillary lymph node metastasis.

## Results

### Expression and Sub-Cellular Distribution of KLF6 in Normal and Tumor Tissues

**Figure 1:**
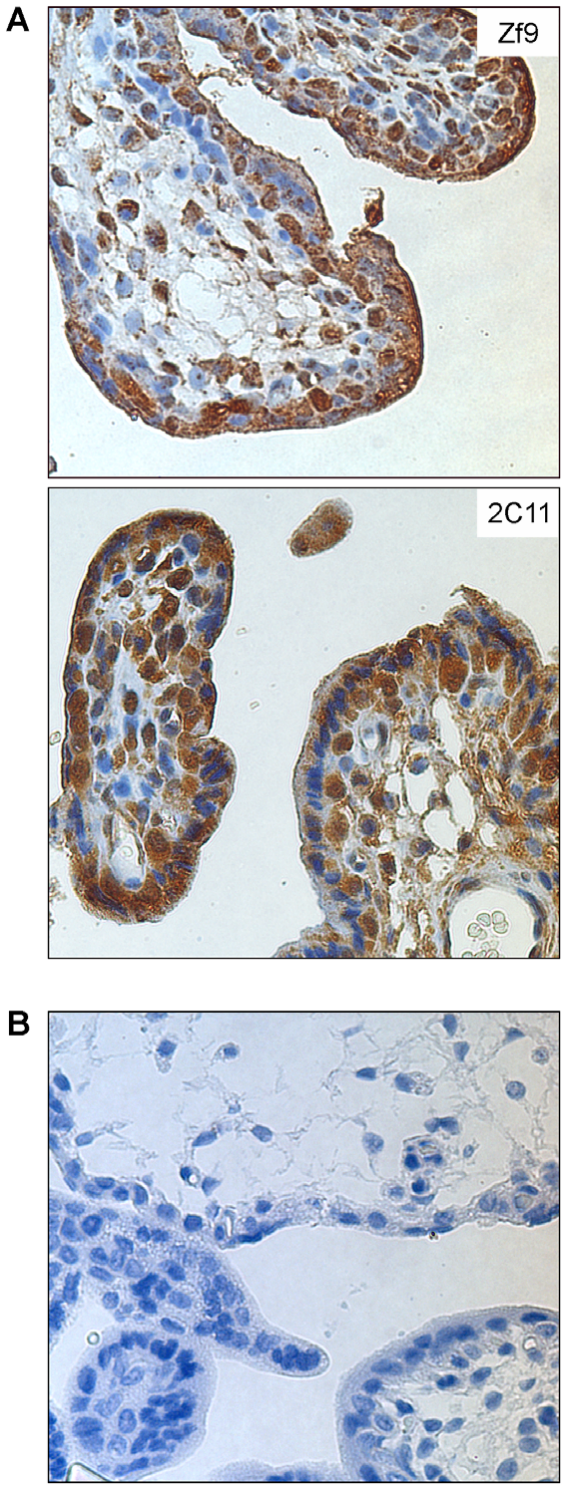
Immunohistochemical assay for KLF6 in placenta tissue section. Immunohistochemistry assays were performed as described in Material and Methods. The specific stain is shown as a brown precipitate and nuclei were blue counterstained with hematoxylin. Magnification is 600×. **A.** Stain pattern obtained for the commercial anti-KLF6 polyclonal antibody (Zf9, top) and for in house produced anti-KLF6 monoclonal antibody (clone 2C11, bottom) in term placenta tissue sections. **B.** Background control of the secondary detection complex EnVision® System Peroxidase Labeled Polymer (Dako, Carpinteira, USA). The placenta tissue section was incubated only with primary antibody dilution buffer, and the secondary detection complex mentioned above.

To determine the pattern of KLF6 protein and its sub-cellular distribution in normal and tumor breast tissues by immunohistochemistry, the specificity of the antibodies was controlled using tissue sections of human term placenta, considering the high level of KLF6 expression in this organ. The specificity of the anti-KLF6 monoclonal antibody (clone 2C11) was previously characterized by immunoprecipitation and Western blot assays [26]. For immunohistochemistry assays, the specificity of this antibody was evaluated with respect to that of the commercial polyclonal *Zf-9* anti-KLF6 antibody. Both antibodies developed similar staining patterns in paraffin-embedded term placenta sections by immunohistochemistry assays, showing nuclear and cytoplasmic localization of KLF6 in peripheral chorionic villous cells, and in inner cells of placenta (Fig. 1A). This result indicates that the specificity of the monoclonal antibody (clone 2C11) is suitable for detection of KLF6 in tissue sections by immunohistochemistry, given similar results than the commercial anti *Zf9* polyclonal antibody. Even similar antibodies staining patterns were found in a breast carcinoma tissue section (Fig. S1).

**Figure 2:**
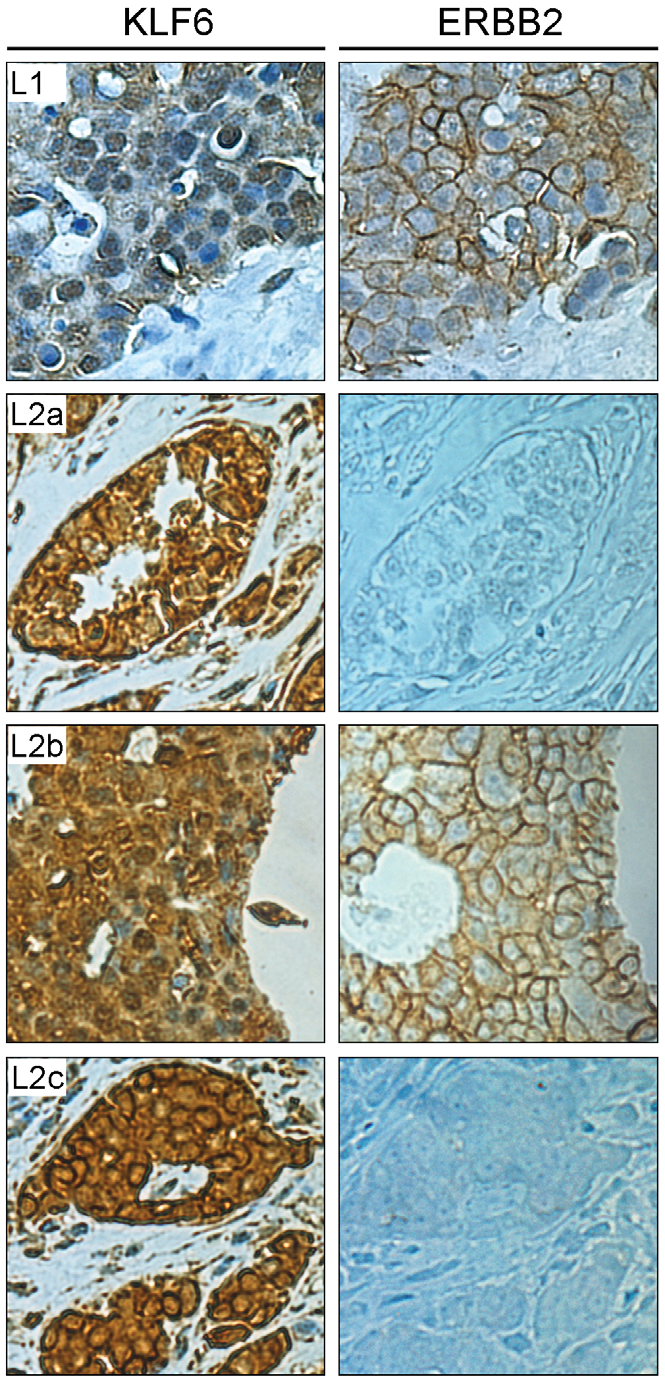
KLF6 and ERBB2 expression in breast cancer tissues. Immunohistochemistry assays in two lots of Tumor tissues Microarray from DAKO (CheckBoard Tissue Tumor human Tissues, T1064, Dako, USA). Tissues were incubated with anti-KLF6 (clone 2C11, *left panel*) or anti-ERBB2 (*right panel*) antibodies. Lot 1 (#00071) is represented in the panel L1, and Lot 2 (#00111) in panels L2a, L2b and L2c. The KLF6 and ERBB2 stains were photographed in the equivalent area of the tissue section at 400× of magnification.

Next, the pattern of KLF6 protein was analyzed by immunohistochemistry assays in several normal and tumor tissue sections derived from paraffin-embedded samples spotted in an array. Results clearly showed a specific and significant staining for KLF6 in both normal and tumor tissues with varied intensity. Also the sub-cellular distribution of KLF6 was confined to the cytoplasm compartment in tumor tissues in contrast with a uniform nuclear and cytoplasmic distribution for each histological normal counterpart (Fig. S2 and Table S1). These results are in good agreement with previous studies showing a ubiquitous expression of KLF6 at the mRNA level [6], [7], and in this work it is shown that substantial amount of KLF6 protein is mainly accumulated in the cytoplasmic compartment of cancer samples. Interestingly, this expression and sub-cellular distribution pattern was not observed in breast cancer tissue sections. In contrast to other type of tumor tissues, KLF6 expression was confined in a large extent and almost exclusively in the nuclear compartment in a high percentage of these breast tumors tissues (Fig. 2, left panel, L1). Similar KLF6 distribution was detected in a different lot of tumor tissue microarrays (Fig. 2, left panel, L2a–c). These results suggest a specific regulation of KLF6 polypeptide in the context of breast tumor, leading to a preferential localization within the nucleus.

ERBB2 expression constitutes a well known marker of breast tumors aggressiveness and its overexpression is associated with a worse prognostic and reduced survival [31]. To assess the relationship of KLF6 protein expression with the malignancy degree of each breast tumor analyzed in this study, the ERBB2 expression level was determined in both lots of tumor tissues microarrays. Interestingly, though ERBB2 expression level varied in different breast tumor samples, tissues having a nuclear localization of KLF6 show higher expression of ERBB2, as observed in both tissues lots (Fig. 2, right panel). It is important to underline that ERBB2 expression was analyzed at the adjacent tissue area where KLF6 was detected.

### Sub-Cellular Localization of KLF6 in Breast Cancer Tissues

As shown previously, nuclear localization of KLF6 observed in cells of breast tumor tissue sections is in line with high expression levels of ERBB2. These results prompted us to extend the analysis of KLF6 cell distribution to a wide breast tumor tissue samples population. Thus, an independent tissue microarray was analyzed, which contains tissues sections from 60 clinical cases comprising an assortment of human breast tumor types (LandMark™). Patient's clinical data, including tumor size, histological grade and stage, and lymph nodes analysis for each case are summarized in Table 1 and detailed by clinical case in Table S2.

**Table 1:** Characteristic of the studied breast carcinomas population.

Parameter	Subcategory	Value
No. of patients		60
Median age, years (range)		65 (31–92)
Tumor stage, n (%)	**I**	17 (28.3)
	II	36 (60.0)
	III	6 (10.0)
	IV	1 (1.7)
Histological grade, n (%)	1	14 (23.3)
	2	34 (56.7)
	3	12 (20.0)
Histopathologic type, n (%)	Ductal	48 (80.0)
	Lobular(1)	6 (10.0)
	Cribiform	2 (3.2)
	Metaplastic	2 (3.2)
	Mucinous	1 (1.7)
	Tubular	1 (1.7)
	Medullary	1 (1.7)
Median tumor size, cm (range)		2.7 (0.7–8.5)
Lymph nodes, n (%)	Negative	11 (18.3)
	Positive	15 (25.0)
	No determined	34 (56.7)

(1) One lobular breast carcinoma tissue was destroyed, and thus not included into the analysis.

**Figure 3:**
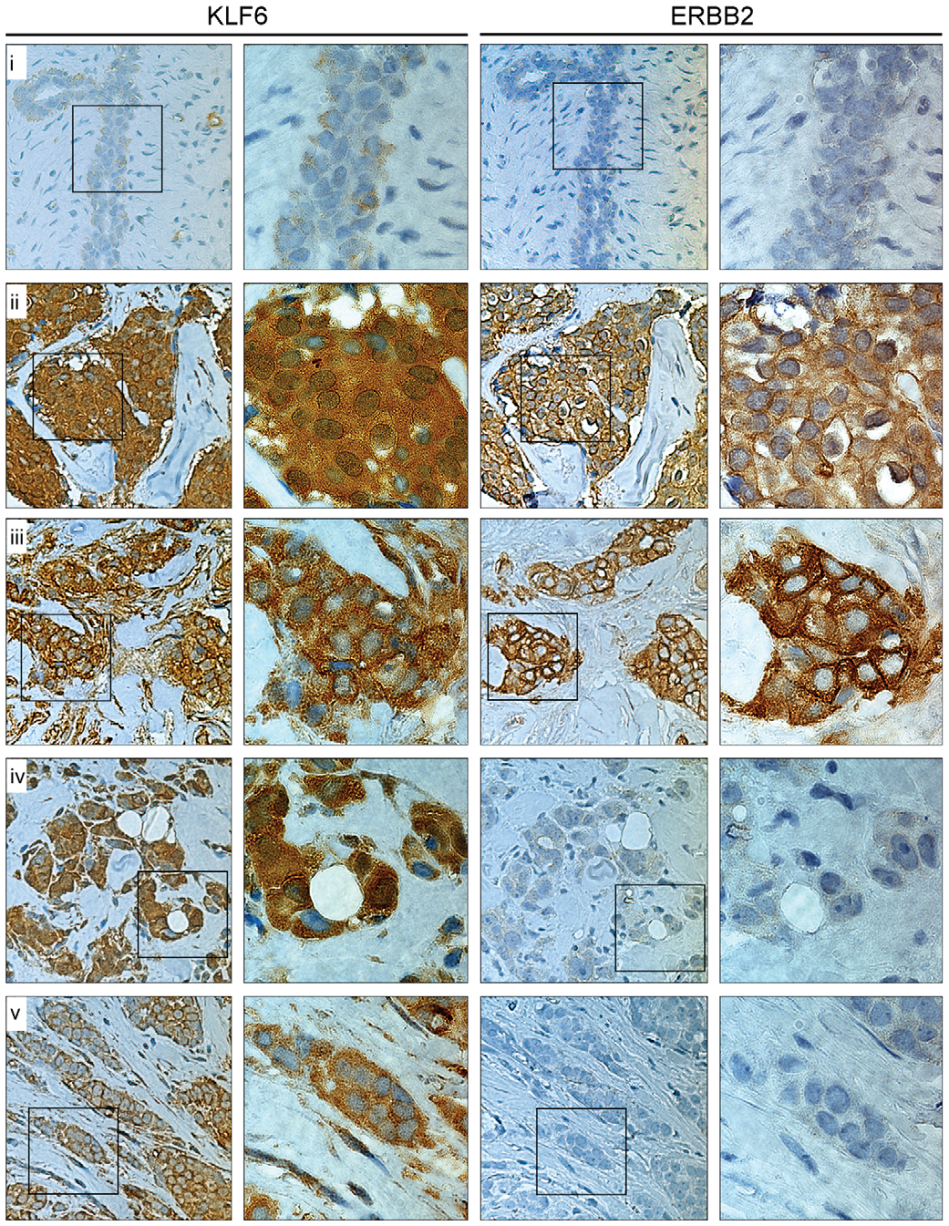
KLF6 and ERBB2 expression in ductal breast cancer tissues. Immunohistochemistry assays in LandMark LD Breast TMA tissue microarray (Ambion, Austin, TX, USA). Tissues were incubated with the anti-KLF6 and anti-ERBB2 antibodies. The left panel of the KLF6 and ERBB2 column pictures has 400× magnification. The square into each 400× picture indicates the area photographed at 1000× and showed in the right panel of each column. Photomicrographs of the immunostain for KLF6 and ERBB2 were taken from equivalent tissue areas. The horizontal panels represents the KLF6 and ERBB2 expression in a fibroadenoma tissue (*i*) and four representative cases for both positive nuclear KLF6 and ERBB2 (*ii*), negative nuclear KLF6 and positive ERBB2 (*iii*), positive nuclear KLF6 and negative ERBB2 (*iv*), and both negative nuclear KLF6 and ERBB2 (*v*). Tissues in *ii–v* correspond to the cases 40, 18, 45, and 6, respectively, described in Tables S2 and S3.

Immunohistochemical assays revealed that epithelial cells of the whole breast tumor tissues population developed a specific staining for KLF6 (Fig. 3 left panel, Table 2 and Table S3). Interestingly, the stain intensity was consistently higher than of a fibroadenoma tissue sample, which expressed low levels of KLF6 that was detected systematically in the cytoplasmic compartment. It is important to remark that no nuclear KLF6 staining was detected in the fibroadenoma breast tissue (Fig. 3 row *i*, left panel; Table S3). This tissue sample was used as control tissue because the architecture and cellular phenotype of a non-pathologic mammary gland tissue are relatively conserved as in normal tissue. Following, the KLF6 immunostaining was also positive in the cytoplasmic compartment of the complete breast tumor tissues population. More importantly, 57.6% (34/59) of the analyzed breast tumors tissues whole population exhibited positive nuclear immunostain for KLF6 (Table 2A). This result is in accordance with the previous observations detailed above.

**Table 2:** Analysis of KLF6 and HER2-ERBB2 proteins in breast tumor tissues.

A. Immunohistochemical analysis for nuclear KLF6 and HER2-ERBB2 in the total breast tumor tissues population		
Protein	Status	Number of tissues (%)
HER2/ERBB2	Not overexpressed	27 (45.8)
	Overexpressed	32 (54.2)
Nuclear KLF6	Positive	34 (57.6)
	Negative	25 (42.4)

B. Correlative analysis of nuclear KLF6 expression and HER2-ERBB2 overexpression			
Nuclear KLF6	HER2/ERBB2	Number of Tissues (%)	Chi-square *p* Value
Positive	Overexpressed	25 (42.4)	0.001
Positive	Not overexpressed	9 (15.3)	
Negative	Overexpressed	7 (11.8)	
Negative	Not overexpressed	18 (30.5)	

### HER2-ERBB2 Expression in the Whole Breast Tumor Tissue Population

Apart of the KLF6 expression analysis, immunohistochemical assays were performed to determine the HER2-ERBB2 aggressiveness marker status in the studied breast tumor population. The positive HER2-ERBB2 cut-off criterion is indicated in Material and Methods. Results indicated that 54.2% (32/59) of the breast tumor population overexpressed HER2-ERBB2 (Table 2A). It is important to mention that abnormal high levels of HER2-ERBB2 are present in approximately 18–20% of breast cancers ([43] and references therein). Nevertheless, many researchers agree that it is amplified and overexpressed in 32% to 55% of DCIS lesions [33], [34], [36]. In this sense, the percentage of HER-ERBB2 overexpressing breast tumor tissues obtained in the present work is in line with the prevalence already reported considering in addition that the tumors population is composed by an assortment of invasive and non-invasive tumor types.

### Significant Correlation of Nuclear KLF6 and HER2-ERBB2 Overexpression

Next, to evaluate the significance between the nuclear KLF6 and HER2-ERBB2 overexpression, immunostain intensities of both proteins were estimated for each breast tumor tissue. Interestingly, a significant subpopulation (chi-square *p* = 0.001, Pearson *p*<0.001) of twenty-five breast tumor tissues overexpressing HER2-ERBB2 were positive for nuclear KLF6 (Table 2B). For the remaining tumor tissues, only sixteen breast tumor tissues were single positive (nine for nuclear KLF6, and seven for HER2-ERBB2), and eighteen were negative for both immunostains (Table 2B).

This analysis clearly suggests that KLF6 may be nuclear localized in aggressive breast tumor tissues determined by the HER2-ERBB2 overexpression status.

### KLF6 Nuclear Localization and ERBB2 Overexpression in Ductal Breast Tumor Tissues

**Figure 4:**
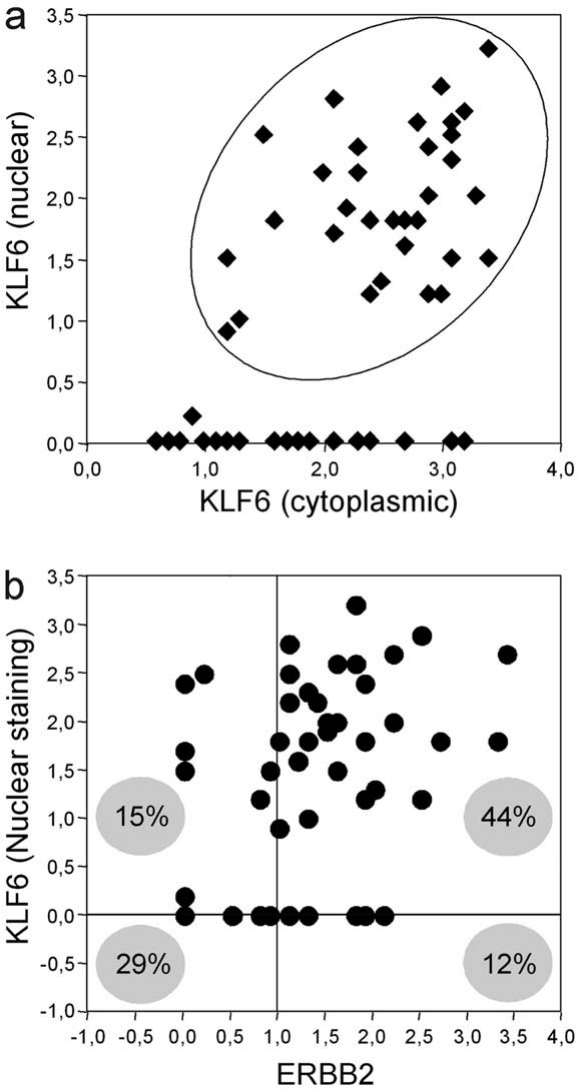
Association between nuclear KLF6 expression and ERBB2-overexpression in ductal breast tumor tissues. The immunohistochemical staining intensity for KLF6 (nuclear and cytoplasmic) and c-erbB2 (cytoplasmic membrane) is represented as scores used a 0.0–4.0 scale estimated over a characteristic cell cluster of each tissue. The scores estimation was obtained as described [50], [51] (see Material and Methods). A score of 1.0 represent less than 10% of positive labeled cells and was set as the cutoff value. **A.** Scatter plot analysis of nuclear KLF6 versus cytoplasmic KLF6. The oval includes positive tissue for both staining patterns. **B.** Scatter plot analysis of nuclear KLF6 versus ERBB2 immunostains. Filled lines settled at 0.0 and 1.0 score indicate cutoff values and partite four populations determined by the KLF6 and ERBB2 staining. The percentage of each population is represented into a gray circle. Especially for the double-negative population (minus 1.0 and 0.0 scores) a point could represent two or more cases.

The tumor tissues microarray included forty-eight ductal breast tumors tissue sections, which represents the major population. Hence, to better define the KLF6 protein sub-cellular localization significance, the analysis was focused on this tissue sub-population. As described in the results section above, the sub-cellular distribution of KLF6 showed a pattern of variable intensity immunostain at the nuclear and cytoplasmic regions, which was referred as immunoscores values (Table S3). A scatter-plot analysis revealed that fifty-eight percent (28/48) of breast ductal tumor tissues express KLF6 both in the nucleus and cytoplasm (Fig. 4A); additionally, ductal breast tumor tissues expressing and non-expressing nuclear KLF6 revealed a significant increase (*p* = 0.005; student *t* test) of the cytoplasmic stain (score mean 2.56 versus 1.99, respectively; see Fig. 4A and Table S3). Regarding HER2-ERBB2 expression, results of immunohistochemical assays showed that fifty-five percent (28/48) of these samples overexpressed ERBB2 protein, giving different stain intensity levels (Fig. 3, right panel, and see Table S3). Within ductal breast tumors, the percentage of ERBB2 overexpressing tissues was almost maintained to those determined for the whole breast tumors population.

Following, the KLF6 nuclear localization was related to the HER2-ERBB2 overexpression in ductal breast tumor tissues. A significant subpopulation of the ductal breast tumor collection [44% (21/48)] showed positive immunostain for nuclear KLF6 and ERBB2 overexpression (Chi-square and Fisher exact tests *p* values equal to 0.005 and 0.003, respectively; Fig. 4B); analysis of the tumor population overexpressing only ERBB2 revealed that the seventy-eight percent (21/27) of ductal breast tumor tissues expressed nuclear KLF6 (Table 3). Moreover, within the tumor population expressing nuclear KLF6, it was found that seventy-five percent (21/28) of the cases overexpressed ERBB2 (Table 3). In contrast, ERBB2-negative ductal carcinomas showed a significant lower KLF6 stain scores (*p* = 0.007; student *t* test) and absence of nuclear staining compared to ERBB2-overexpressing ductal carcinomas (score mean 1.9 versus 2.5, respectively; Table S3). In agreement with this observation, a similar direct correlation between the KLF6 and ERBB2 expression was found at the RNA level of the *klf6* and *HER2/neu* –encoding genes in an independent study of breast tumors population, as determined by RNA microarray hybridization (Fig. S3).

**Table 3:** Analysis of the nuclear KLF6 expression along with the Estrogen receptor alpha status and HER2-ERBB2 overexpression in breast tumor tissues.

A. Immunohistochemical analysis for Estrogen Receptors alpha status		
Breast tumors population	Estrogen Receptor Status	Number of tissues (%)
Total	Positive	38 (63.3)
	Negative	21 (35.0)
	ND	1 (1.7)
Ductal	Positive	30 (62.5)
	Negative	17 (35.4)
	ND	1 (2.1)
ND: None determined		

B. Nuclear KLF6 expression and ERBB2 overexpression versus Estrogen Receptors alpha status in ductal breast tumor tissues			
Nuclear KLF6/ERBB2	Total Number of tissues	Estrogen Receptor	Number of Tissues (%)
Positive/Overexpressed	21	Positive	15 (71.4)
		Negative	6 (28.6)
Positive/Not overexpressed*	7*	Positive	5 (71.4)
		Negative	1 (14.3)
Negative/Overexpressed	6	Positive	3 (50.0)
		Negative	3 (50.0)
Negative/Not overexpressed	14	Positive	7 (50.0)
		Negative	7 (50.0)

(*)One tissue was not assessed for Estrogen Receptor alpha status due to it was destroyed.

Overall these observations clearly suggest that the nuclear expression of KLF6 frequently occurs in ERBB2-overexpressing ductal breast tumor cases, which is in line with tumor aggressiveness. In addition, regardless its sub-cellular distribution, the global *klf6* gene expression seems to be augmented in accordance with the *HER2/neu* gene expression level in this type of ERBB2-overexpressing ductal breast tumors.

### Estrogen Receptor Alpha Status

To validate our previous results regarding the nuclear distribution of KLF6 in ERBB2-overexpressing breast tumors the expression of Estrogen Receptor alpha was determined as an additional established risk factor for breast cancer. It is well known that Estrogen Receptor alpha are expressed in up to 70% of all breast cancers [31].

Consistently with this, thirty-eight (63.3%) tissues of the total breast tumor population were positive for Estrogen Receptor alpha. Similar results were observed in the ductal breast tumor subpopulation, where thirty tissues (62.5%) were positive for Estrogen Receptor alpha (Table 3A).

Following we examined the Estrogen Receptor alpha-α status depending on nuclear KLF6 distribution and ERBB2-overexpression in ductal breast tumor tissues. In this regard, seventy-one percent (20/28) of nuclear KLF6 positive tissues were also positive for Estrogen Receptor alpha; in comparison, sixty-four percent of ERBB2-overexpressing tissues also expressed Estrogen Receptor alpha. More importantly, seventy-one percent (15/21) of the ductal breast tumor tissues sub-population with both nuclear KLF6 distribution and ERBB2-overexpression also were positive for Estrogen Receptor alpha (Table 3B). This finding is of great importance because this result indicates high correlation of the nuclear expression of KLF6 in ductal breast tumor tissues with other established risk factor, and also together with ERBB2.

### Clinico-Pathological Parameters

To determine the relationship of nuclear KLF6 distribution in ductal carcinomas, the clinico-pathological parameters were analyzed in those tumor tissues overexpressing ERBB2. TNM staging system criteria respect to the size and tumor stage, and analysis of axillaries lymph nodes was used for evaluation of clinical provided data [44]. The ERBB2 overexpressing ductal breast carcinomas were analyzed with respect to size, tumor stage and histological grade, presence or absence of axillary lymph node metastasis, and nuclear KLF6 immunostain.

As described in Table 4, the ductal breast tumor population is mainly contained in small and medium tumors sizes [(23/27, 85%) with <2.0 cm and 2.1–5.0 cm, respectively], and stage I and II tumors (24/27, 89%). KLF6 is preferentially expressed in 91% (10/11) of small ERBB2 overexpressing ductal breast tumors, while the expression percentage is homogeneous in the remaining tumor size subpopulation. Similar results were obtained with respect to tumor stages and histological grades. In this regard, 86% (6/7) of stage I-ERBB2 overexpressing ductal breast tumors have positive nuclear stain for KLF6. A similar pattern was observed for stage II-ERBB2 overexpressing tumors though in a lower proportion (76%; 13/17).

**Table 4:** Clinicopathological parameters of ERBB2-overexpressing ductal breast tumors and nuclear KLF6.

Parameter	Subcategory	Positive KLF6 (%)	Negative KLF6 (%)	Number of Cases	Chi-square p value
Size	<2.0 cm	10 (91)	1 (9)	11	0.385
	2.1–5.0 cm	8 (67)	4 (33)	12	
	>5.1 cm	1 (50)	1 (50)	2	
	ND	2 (100)	0 (0.0)	2	
Stage	I	6 (86)	1 (14)	7	0.694
	II	13 (76)	4 (24)	17	
	III	1 (50)	1 (50)	2	
	IV	1 (100)	0 (0.0)	1	
Histological grade	1	7 (100)	0 (0.0)	7	0.046
	2	9 (60)	6 (40)	15	
	3	5 (100)	0 (0.0)	5	
Lymph nodes	Positive	7 (60)	1 (40)		0.177
	Negative	3 (50)	3 (50)		
	ND	11 (75)	2 (25)		
Total tissues		21 (78)	6 (22)	27	

ND: No determined.

Histological grades of tumors were also analyzed. ERBB2 overexpressing ductal breast tumors did not revealed a preferential relationship of histological grades and nuclear KLF6 expression. In this sense, nuclear distribution of KLF6 was observed in 100% (7/7) in ERBB2 overexpressing ductal breast tumors with histological grade 1. Nevertheless, similar results were found for tumors with histological grades 2 and 3 (60%, 9/15; 100%, 5/5, respectively; Table 4). Due to the relative low number of tumor cases, only for histological grade was possible to establish a significant correlation by chi square statistical analysis, and non significant statistical results were obtained respect to the size and stages of the breast tumor cases (see Table 4). Similar statistical results were obtained by analyzing axillary lymph nodes metastasis.

Fifty-two percent (14/27) of patients included in the study population overexpressing ERBB2 were analyzed for metastatic lymph nodes (Table 4). Interestingly, the greater percentage of metastatic (positive) lymph nodes occurred in patients with ERBB2 overexpressing ductal breast tumors showing in addition positive nuclear stain for KLF6 (88%, 7/8), and represent the major subpopulation of cases with analyzed axillary lymph nodes. Negative lymph nodes were equally distributed between positive or negative KLF6 nuclear stained, ERBB2 overexpressing tumors (Table 4).

It is important to mention that the low number of analyzed ductal breast tumor cases is not enough for statistical analysis. Nevertheless, the obtained results suggest that a high percentage of ERBB2-overexpressing ductal carcinomas may express KLF6 at the nuclear compartment during early stages of development. In addition, patients harboring ductal breast carcinomas corresponding to the analyzed population seem to be prone to develop metastasis, in order to the prevalent percentage of positive lymph nodes in patients affected by this tumor type.

## Discussion

Expression and sub-cellular distribution of KLF6 was determined in normal and tumor tissues by using tissue microarrays and immunohistochemistry with two specific antibodies. In accordance with previous reports, we found a ubiquitous expression of KLF6 either in normal and tumor tissues [5], [6], [7]. In this regard, KLF6 sub-cellular distribution is mainly nuclear in normal tissues in contrast with an entirely cytoplasmic expression in the tumor counterpart (Fig. S2 and Table S1). This expression pattern completely matches with the role as tumor suppressor described for KLF6 since mutations of *klf6* gene were associated to different human neoplasms [9], [10], [11], [12], [13], [14], [15], [45]. For instance, it is possible to hypothesize that absence of a nuclear expression of KLF6 prevents the regulation of specific target genes involved in negative control of cell growth.

Despite of the expression pattern tendency described above, the KLF6 expression was found markedly changed in a significant percentage of breast tumor tissues, where it was mostly detected in the nucleus of ERBB2-overexpressing carcinoma cells (Fig. 2). Breast cancer represents a heterogeneous disease in oncology and the ERBB2 status has become a routine prognostic and predictive factor in standard measurements in the management of patients together with Estrogen Receptor alpha, which are expressed in up to 70% of all breast cancers [31], [32]. *HER-2/neu* oncogene encodes a member of the epidermal growth factor receptor (EGFR) family of tyrosine kinases and is located on chromosome 17q21. Abnormal high levels of ERBB2 are present in approximately 18–20% of breast cancers and gene amplification is the predominant mechanism leading to ERBB2 overexpression which in turn enhance cell proliferation ([43] and references therein).

KLF6 expression and sub-cellular distribution was further analyzed in breast cancer samples, mainly focused on ductal breast tumor tissue cases. Concerning to the ERBB2 status, many researchers agree that it is amplified and overexpressed in 32% to 55% of DCIS lesions. Moreover, *HER-2/neu* gene amplification and overexpression in ductal tumors is more common in higher grade rather than in lower grade lesions, and also has been associated with aneuploidy and higher proliferative indexes [33], [34], [36]. In focus on the ERBB2-positive ductal breast tumor population, the results presented in this work demonstrated that there is a significant percentage of tissues that express nuclear KLF6 (Fig. 3 and Table 2). Interestingly, this expression pattern was also found highly correlated with a positive stain for Estrogen Receptor that represents other established risk factor, and thus further validates our results (Table 3B). An independent analysis performed by RNA microarrays hybridization of samples obtained from a distinct breast tumor group indicated a direct correlation between the expression level of *klf6* and *HER-2/neu* genes (Fig. S3). In addition, KLF6 knockdown in MCF7 breast cancer cells leads to a cell cycle arrest affecting the G1-S transition (Table S4). Thus, despite of the limited knowledge about the KLF6 role in breast cancer, our results strongly indicate that KLF6 is expressed in breast tumor tissues, even at higher levels than a control breast tissue (Fig. 3, Table 2, and Table S3). Nuclear localization of KLF6 together with an ERBB2-overexpression suggests that KLF6 function is associated with the malignant phenotype, grow and development of this type of breast tumor.

Though KLF6 is a transcription factor having a conserved nuclear localization signal and the analysis of its primary amino acid sequence predicts a nuclear localization of this protein, KLF6 is frequently detected in the cytoplasm of several tissues and cell lines as in HEK and Cos-7 cells (Slavin et al., 2004). However, KLF6 is mainly located in the nucleus in other cell line like A 549 derived from lung carcinoma (V. Andreoli and J.L. Bocco, unpublished). Similar results showing nuclear and/or cytoplasmic distribution was observed for endogenous KLF6 in normal and tumor tissues by immunohistochemistry ([46]; this work). Following these results, it is interesting to hypothesize that some endogenous or external signals, still not known, could regulate KLF6 distribution in specific cells and in some ones, like in ductal breast cancer cells; these signals could be constitutively activated. In this sense, cell stimulation mediated by phorbol esters alone or in combination with ionomycin, led GFP-tagged KLF6 through the nuclear compartment [26]. Additionally, endogenous KLF6 translocated to the nucleus upon activation of hepatic stellate cells and denudation of endothelial HUVEC cells [47], [48]. Thus, the dynamics of nuclear localization of KLF6 seems to be finely tuned in normal cells; alterations of this regulatory mechanism in the context of ductal breast cancer cells might promote constitutive KLF6 expression within the nucleus which is in direct association with a malignant phenotype, as shown here.

Alternatively, the half life of cytoplasmic KLF6 protein could be shorter due to a mechanism still to be revealed. However, independently of which mechanism takes place, it was recently shown that reduction or absence of KLF6 abrogates the negative control of breast cancer cell proliferation triggered by Estrogen Receptor alpha through the signaling pathway mediated by c-Src and Akt activation [49]. Thus, in addition to its nuclear localization and its function as a transcription factor, cytoplasmic KLF6 is able to interact with c-Src protein and thereby interferes with Estrogen Receptor alpha-mediated cell growth of breast cancer cells [49].

In regard to nuclear KLF6, it could have permanent accessibility to specific gene promoters in the context of the chromatin structure of these particular cells. The knowledge of the complete set of target genes regulated by KLF6 in ductal breast cancer is a huge task to be undertaken to gain further insights about KLF6 in this pathology.

There is a continual drive to identify markers that will aid in predicting prognosis and response to therapy of breast carcinomas. They are also fundamental for categorizing patients into different prognostic groups, when combined with information regarding tumor size, grade, lymph-vascular invasion and nodal stage (reviewed in [31], [32], [33], [34], [36]). To further understand the impact of nuclear expression of KLF6 in ERBB2-overexpressing ductal breast tumors we compare this stain pattern respect to clinico-pathological parameter such as tumor stage, histological grade and size, and lymph node involvement. Given its histological characteristics, DCIS breast tumors have no metastatic potential, but from 1% to 2% of patients with a diagnosis of pure ductal breast tumor subsequently develop metastases either because of occult invasion or because of the progression of unsuspected residual disease to invasive breast cancer [36]. In this sense, ERBB2 detection is associated with high-grade tumors, lymph node involvement, a higher rate of disease recurrence and mortality, and thus a poor prognosis [31]. Although we have analyzed a small number of cases, in this report we further suggest that KLF6 is nuclear expressed in a significant percentage of ERBB2-overexpressing ductal breast tumors, and this nuclear stain pattern may be related to the malignancy and aggressiveness together with ERBB2-overexpression in early stage and small ductal breast tumors because of the presence of metastatic event in lymph nodes of patient harboring this tumor type.

Although is still early to fully understand the KLF6 function in tumorigenesis, results of this work suggest that the tumor suppressor function described for KLF6 could be recruited and/or subverted in the context of breast cancer environment, contributing to tumor development.

This report represents the first step of an effort to elucidate the KLF6 function within the breast carcinoma environment, essential to understand the contribution of KLF6 for breast cancer pathogenesis and also as a new molecular marker candidate for prognosis and therapy.

## Materials and Methods

### Tissue Procurement

Checkerboard Multi-Tumor and Multi-Normal Tissue micro-Array containing paraffin-embedded normal or tumor tissues samples of multiple human organs and placenta were purchased from Dako, Carpinteria (CA, USA). The KLF6 expression in breast tumors tissues was determined in LandMark LD Breast TMA tissue array, which contain tissue sample from 60 breast cancer cases (Ambion, Austin, TX, USA). General information of patient and tumor features as age, sex, axillaries lymph nodes status, tumor type, stage, and size are summarized in the Table 1 and Table S2.

### Immunohistochemistry Assay

The tissue micro-Arrays were deparaffinized by treating twice with xylene for 5 min and hydrated with serial aqueous-increased ethanol solutions. Heat-induced epitope retrieval of deparaffinized tissues was performed by using Target Retrieval Solution (Dako, CA, USA) at a temperature range of 95–99°C for 20 min and following blocked with 10% inactivated Fetal Calf Serum in PBS for 30 min at room temperature. Endogenous peroxidase tissue activity was blocked with Peroxidase Blocking Reagent (Dako, CA, USA) for 5 min at room temperature. Blocked tissues were incubated overnight at 4°C in a humidified chamber with a suitable dilution of primary antibody in Antibody Diluent with Background Reducing Component Solution (Dako, CA, USA). Non-attached primary antibody was removed by washing three times with PBS avoiding tissue drying. The immunodetection was performed by incubating the tissues with Envision System Peroxidase Labeled Polymer (Dako, CA, USA) for 30 min at room temperature. Diaminobenzidine (DAB; Dako, CA, USA) was used for chromogenic reaction and Mayer's hematoxylin (Lillie's Modification; Dako, CA, USA) for nuclear counterstain.

### Antibodies

Immunohistochemistry assays were performed with an anti-KLF6 mouse monoclonal antibody (clone 2C11, 1∶150) whose specificity was previously determined [26], or using the commercial anti-KLF6 polyclonal antibody (anti-Zf9; R-173, 1∶50; Santa Cruz Biotech., USA). Term placenta tissue sections (Dako, Carpinteira, CA, USA), where endogenous KLF6 is highly expressed, were used to control the specificity of anti-KLF6 antibodies and Envision System Peroxidase Labeled Polymer (Dako, Carpinteira, CA, USA, Fig. 1A and 1B, respectively). A breast tumor tissue section (Dako, Carpinteira, CA, USA) was also used for checking antibodies staining patterns (Fig. S1). The expression of ERBB2 was determined with a rabbit polyclonal antibody anti-human c-erbB2 oncoprotein (1∶200; A0485, Dako Carpinteira, USA), widely used for immunohistochemistry tests in patients [31].

### Estrogen Receptor Alpha

The Estrogen Receptor alpha status of breast tissue samples was determined at a private clinical diagnosis institute by immunohistochemical staining using the automated system Dako Autostainer Universal Staining. Epitope retrieval was induced by microwave heating using 0.01 M citrate retrieval solution (pH 6.0).

Immunohistochemistry assays for Estrogen Receptor alpha detection were performed with an anti-Estrogen Receptor alpha mouse monoclonal antibody (clone ER 6F11, NCL-L-ER-6F11, 1∶100, Novocastra, UK). The immunodetection was performed by incubating the tissues with Dako LSAB+ System-HRP (Dako, CA, USA). Estrogen Receptor alpha expression was considered positive when at least 10% of the tumor cells showed uniform nuclear staining (reviewed in [31]).

### Immunoscoring

The immunohistochemical stain intensity of individual cells was scored on a scale of 0 to 4, representing no staining (0) to highest staining intensity (4), from a representative cell cluster of each tissue. To calculate immunoscores, the proportion of cells (percentage) at each immunostain intensity was multiplied by the corresponding intensity value and these products were added to obtain an immunostain score ranging from 0 to 4 as previously described [50], [51]. For KLF6, the immunostain intensity was evaluated in both nucleus and cytoplasm cell compartment. HER-2/ERBB2 overexpression was considered positive when the 30% (staining score higher than 1.0) of the tumor cells showed uniform intense membranous staining according to the recommendations of The American Society of Clinical Oncology/College of American Pathologists (ASCO/CAP) published in January 2007 [52]. The immunostaining intensity was blindly and independently defined by two individual investigators. Discordances were reconciled with a new stain intensity definition and the obtained scores were averaged.

### Statistical Analysis

Association between nuclear KLF6 localization and HER-2/ERBB2 as one of the most effective breast tumor aggressiveness marker as well as patients' characteristics and histological parameters within the tissue population was assessed using contingency tables analysis (Chi-square X^2^ test) including Yate's continuous correction, and validated with Fisher's exact test. Differences within immunostain intensity scores among nuclear or cytoplasmic mark for KLF6 and cytoplasmic membrane for ERBB2 were evaluated by using student *t* test. Significant differences were considered as *P*<0.05 (two-tails).

## Supporting Information

Figure S1 Experimental control of immunohistochemitry assays in a breast tumor tissue. Control experiment of the stain pattern obtained without primary antibody for background control (non-antibody, left), with the polyclonal (anti-Zf9) or with the monoclonal (clone 2C11) anti-KLF6 antibodies (middle and right panels respectively; see also legend to Fig. 1). The blue stain is due to hematoxylin as a nuclei counterstain. Upper panel shows pictures taken at 200× magnification. The square into each 200× picture indicates the photographed area at 1000× that are shown at the lower panel, as indicated. Photomicrographs of the immunostain for KLF6 were taken from equivalent tissue areas.
(4.80 MB TIF)

Figure S2 KLF6 immunostain in normal tissue and its homologous tumoral-derived specie. Immunohistochemical assays for KLF6 using Multi-Tumor and Multi-Normal Tissue micro-Array (TMA) incubated with clone 2C11 monoclonal antibody as described in legend to Fig. 1. The represented organ is mentioned on the top for normal (upper panel) and tumor (lower panel) status. Magnification: 1000×. Specific stain is shown in brown and nuclei in blue, counterstained with hematoxylin.
(4.49 MB TIF)

Figure S3 klf6 and HER2/neu genes expression in breast tumor tissues. The expression of both klf6 and HER-2/neu genes were analyzed from existing microarray data for 21 human breast cancer tissue samples corresponding to HER2/neu non-amplified tumors. The expression of each gene is represented as arbitrary units (AU). A. Two groups were defined with differential expression levels. The two groups were discriminated by a cut-off value represented by an ERBB2 expression level of 800 AU. An oval enclose each group name as Group 1 (upper oval) and Group 2 (lower oval). B. Group 1: breast cancer tissues with ERBB2 levels above 800 AU (Pearson's R: 0.713). C. Group 2 with ERBB2 levels below 800 AU (Pearson's R: 0.706).
(0.15 MB TIF)

Table S1 KLF6 expression and sub-cellular distribution in normal and tumor tissues. Stain intensity as null (−) to strong (++++) NA: no available tissue.
(0.04 MB DOC)

Table S2 Individual patients' clinical characteristics and immunohistochemical analysis for KLF6 and ERBB2. Tumor size is represented in centimeters (cm). Patient's age represented in years. pT: staging designation representing size and infiltration of the tumor. pN: lymph node involvement (0: no involvement/1 lymph nodes involved). pN = X: none analyzed lymph nodes. Total lymph nodes: number of nodes tested. Positive nodes: number of nodes showing cancer metastasis. (*) destroyed tissue.
(0.12 MB DOC)

Table S3 Individual patients' immunohistochemical analysis for KLF6, ERBB2 and Estrogen receptors. KLF6 (nuclear and cytoplasmic) and ERBB2 (cytoplasmic membrane) stain intensity are represented as score scale (0.0–4.0). (+)/(−): indicates if nuclear KLF6 and ERBB2 was considered positive or negative, respectively. (−−) not specified parameter. ND: None determined.
(0.14 MB DOC)

Table S4 Cell Cycle status of MCF-7 transfected with KLF6 specific siRNA. MCF-7 cells were transfected with scrambled (SCR) or KLF6 specific siRNAs (A and B) as indicated (see also [25]). For flow cytometric cell cycle analysis cells were harvested after 72 h, fixed in 70% ethanol, treated with RNase A (125 mg/ml) and propidium iodide (20 ug/ml), analyzed using a cytometer apparatus and evaluated with Cylchred software. The experiment was conducted in triplicates. Statistical analysis was performed using a one-way ANOVA-Bonferroni analysis where p values<0.05 were considered significant.
(0.03 MB DOC)
