# Supplementary figures and images for: Nuclear Expression of KLF6 Tumor Suppressor Factor Is Highly Associated with Overexpression of ERBB2 Oncoprotein in Ductal Breast Carcinomas
Source: PLoS One. 2010 Jan 28;5(1):e8929. doi: 10.1371/journal.pone.0008929 (PMC2812494; doi:10.1371/journal.pone.0008929)

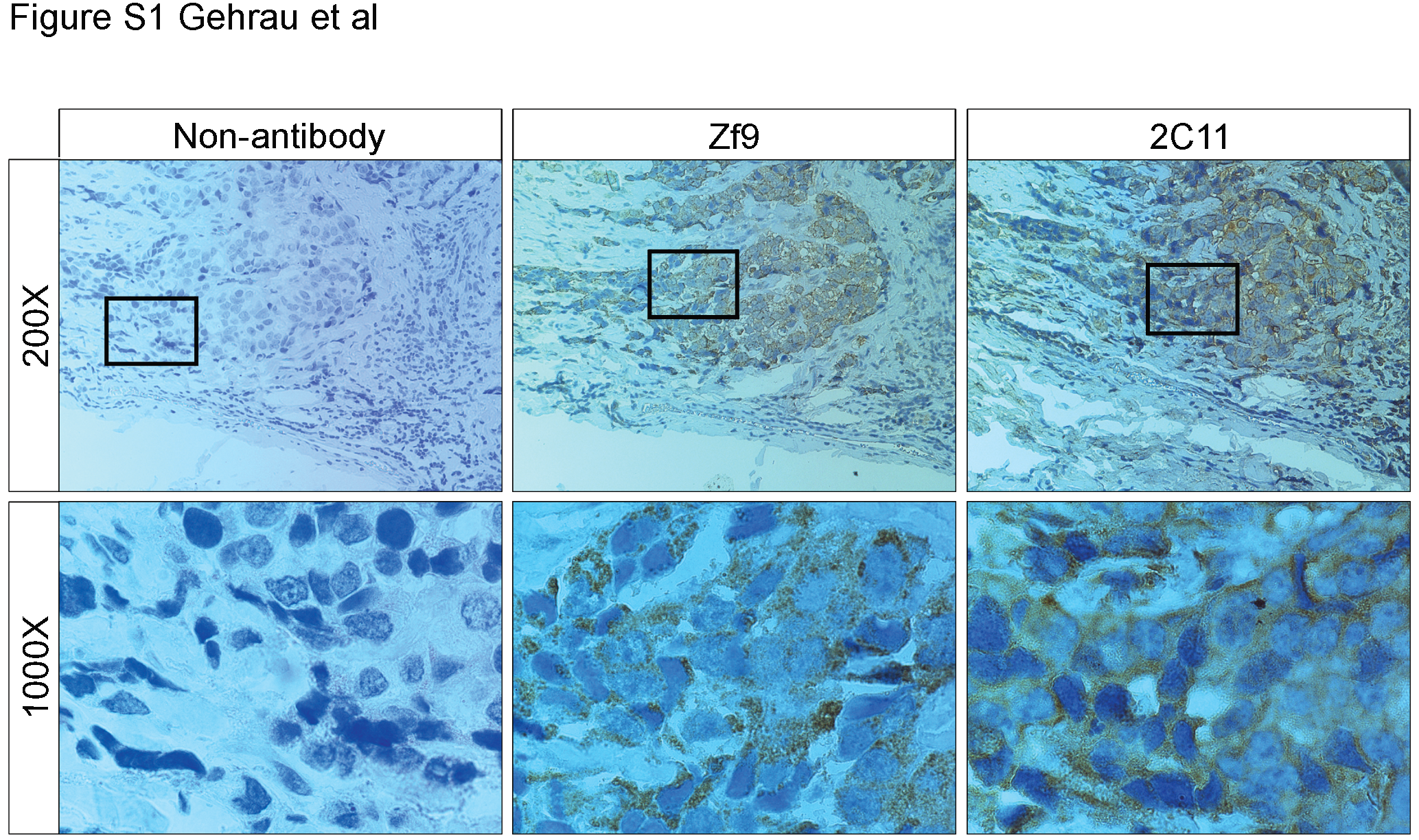

Supplement: Figure S1 — Experimental control of immunohistochemitry assays in a breast tumor tissue. Control experiment of the stain pattern obtained without primary antibody for background control (non-antibody, left), with the polyclonal (anti-Zf9) or with the monoclonal (clone 2C11) anti-KLF6 antibodies (middle and right panels respectively; see also legend to Fig. 1). The blue stain is due to hematoxylin as a nuclei counterstain. Upper panel shows pictures taken at 200× magnification. The square into each 200× picture indicates the photographed area at 1000× that are shown at the lower panel, as indicated. Photomicrographs of the immunostain for KLF6 were taken from equivalent tissue areas. (4.80 MB TIF) [file pone.0008929.s001.tif]

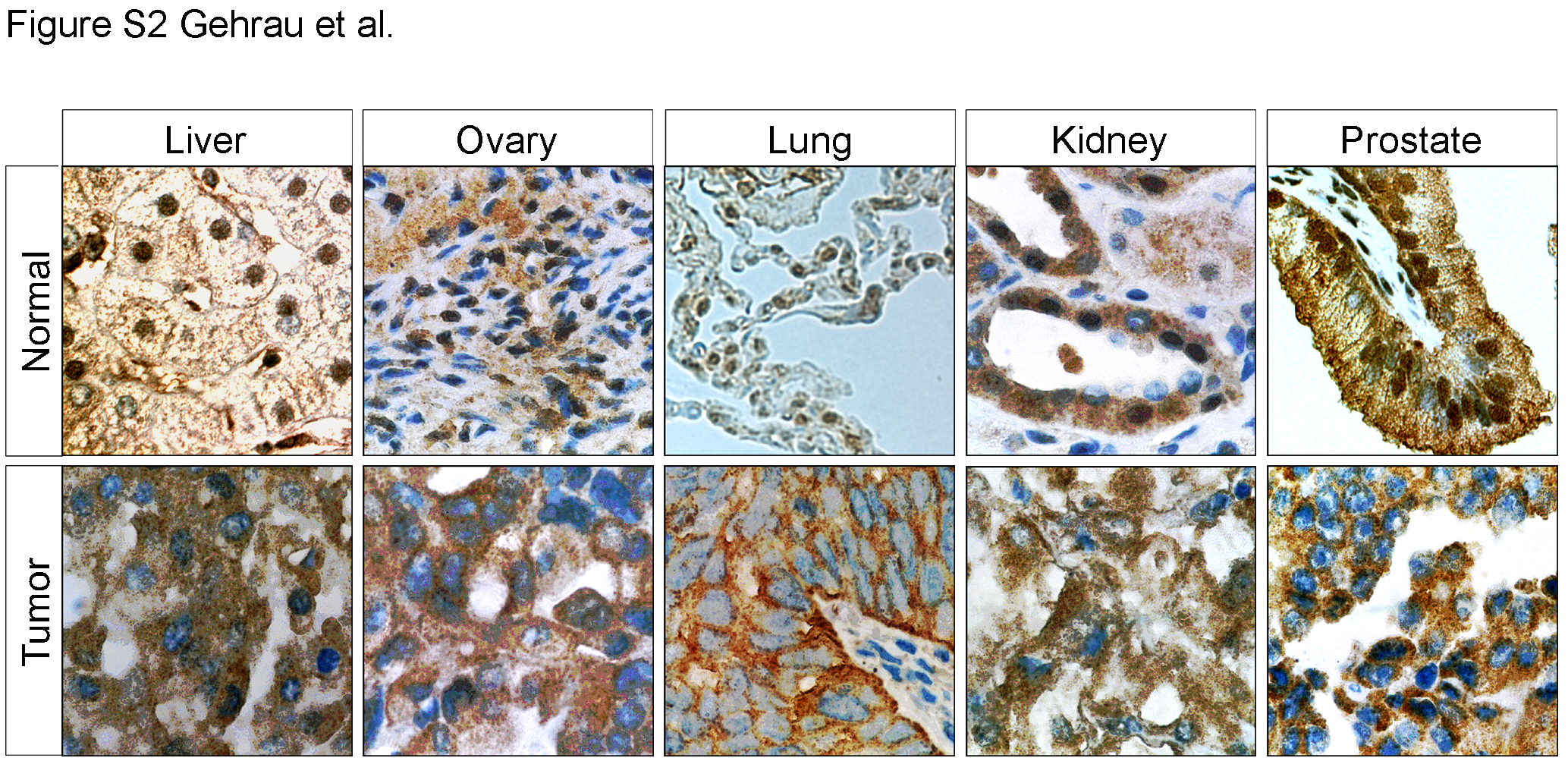

Supplement: Figure S2 — KLF6 immunostain in normal tissue and its homologous tumoral-derived specie. Immunohistochemical assays for KLF6 using Multi-Tumor and Multi-Normal Tissue micro-Array (TMA) incubated with clone 2C11 monoclonal antibody as described in legend to Fig. 1. The represented organ is mentioned on the top for normal (upper panel) and tumor (lower panel) status. Magnification: 1000×. Specific stain is shown in brown and nuclei in blue, counterstained with hematoxylin. (4.49 MB TIF) [file pone.0008929.s002.tif]

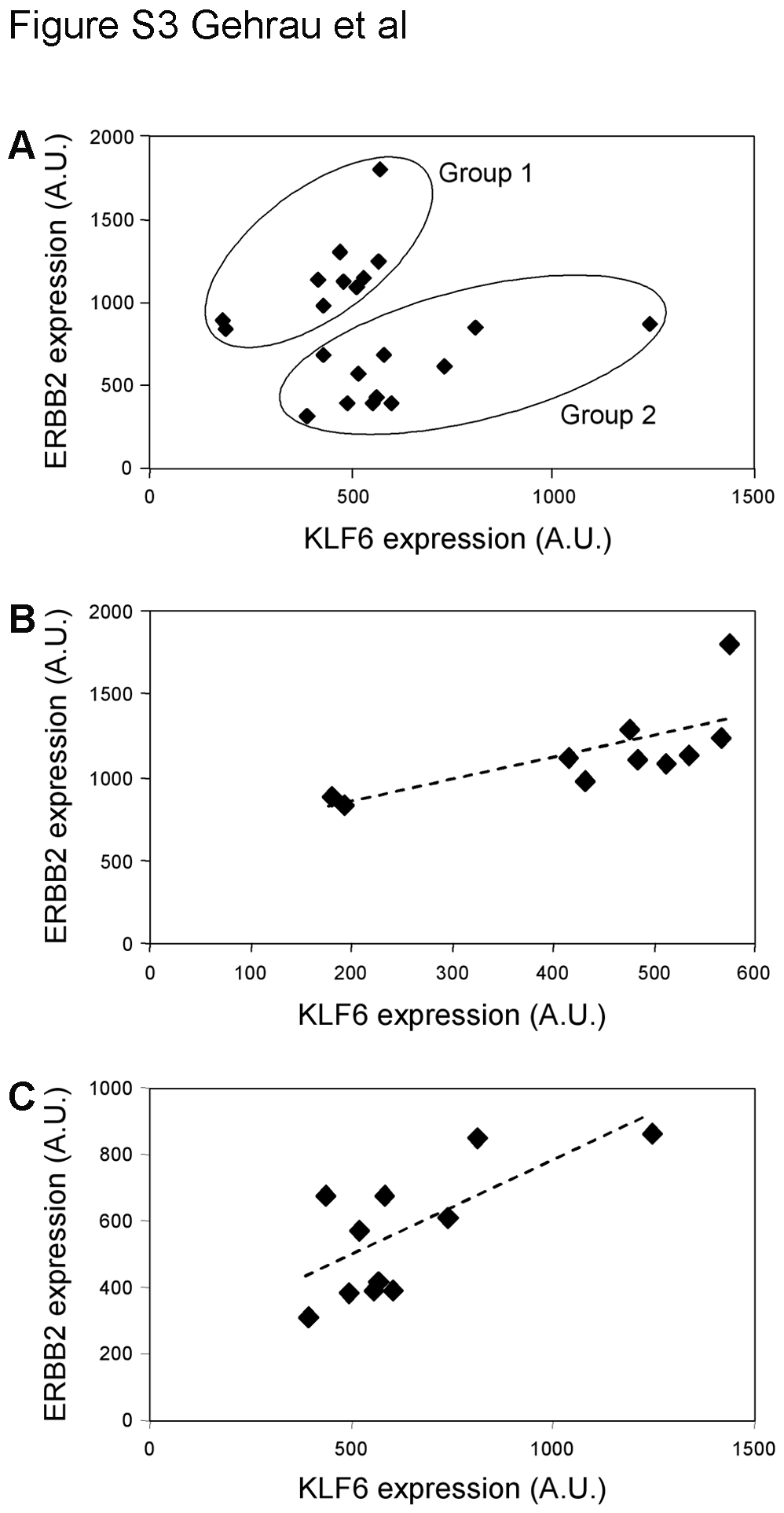

Supplement: Figure S3 — klf6 and HER2/neu genes expression in breast tumor tissues. The expression of both klf6 and HER-2/neu genes were analyzed from existing microarray data for 21 human breast cancer tissue samples corresponding to HER2/neu non-amplified tumors. The expression of each gene is represented as arbitrary units (AU). A. Two groups were defined with differential expression levels. The two groups were discriminated by a cut-off value represented by an ERBB2 expression level of 800 AU. An oval enclose each group name as Group 1 (upper oval) and Group 2 (lower oval). B. Group 1: breast cancer tissues with ERBB2 levels above 800 AU (Pearson's R: 0.713). C. Group 2 with ERBB2 levels below 800 AU (Pearson's R: 0.706). (0.15 MB TIF) [file pone.0008929.s003.tif]
