# Supplementary material for: Nuclear Expression of KLF6 Tumor Suppressor Factor Is Highly Associated with Overexpression of ERBB2 Oncoprotein in Ductal Breast Carcinomas
Source: PLoS One. 2010 Jan 28;5(1):e8929. doi: 10.1371/journal.pone.0008929 (PMC2812494; doi:10.1371/journal.pone.0008929)
Supplement: Table S1 — KLF6 expression and sub-cellular distribution in normal and tumor tissues. Stain intensity as null (−) to strong (++++) NA: no available tissue. (0.04 MB DOC) [file pone.0008929.s004.doc]

Table S1. KLF6 expression and sub-cellular distribution in normal and tumor tissues

| Tissue | Normal |  | Tumor |  |
| --- | --- | --- | --- | --- |
|  | Nucleus | Cytoplasm | Nucleus | Cytoplasm |
| Thyroid | ++ | ++ | - | ++++ |
| Lung | +++ | + | - | ++++ |
| Liver | ++ | ++ | - | +++ |
| Kidney | +++ | ++ | - | +++ |
| Colon | + | + | - | +++ |
| Ovary | ++ | + | - | +++ |
| Pancreas | - | + | - | +++ |
| Prostate | +++ | ++++ | - | ++++ |
| Uterus | - | + | ++ | + |
| Brain | - | ++ | - | + |
| Lymph node | +++ | +++ | ++ | ++++ |
| Seminal vesicle | +++ | ++++ | N/A | N/A |
| Salivary gland (Submandibular) | - | ++ | N/A | N/A |
| Salivary gland (Parotid) | - | + | N/A | N/A |
| Tonsil | + | +++ | N/A | N/A |
| Stomach | - | + | - | + |

Stain intensity as null (-) to strong (++++)

NA: no available tissue.
