# Supplementary material for: Nuclear Expression of KLF6 Tumor Suppressor Factor Is Highly Associated with Overexpression of ERBB2 Oncoprotein in Ductal Breast Carcinomas
Source: PLoS One. 2010 Jan 28;5(1):e8929. doi: 10.1371/journal.pone.0008929 (PMC2812494; doi:10.1371/journal.pone.0008929)
Supplement: Table S2 — Individual patients' clinical characteristics and immunohistochemical analysis for KLF6 and ERBB2. Tumor size is represented in centimeters (cm). Patient's age represented in years. pT: staging designation representing size and infiltration of the tumor. pN: lymph node involvement (0: no involvement/1 lymph nodes involved). pN = X: none analyzed lymph nodes. Total lymph nodes: number of nodes tested. Positive nodes: number of nodes showing cancer metastasis. (*) destroyed tissue. (0.12 MB DOC) [file pone.0008929.s005.doc]

Table S2. Individual patients’ clinical characteristics and immunohistochemical analysis for KLF6 and ERBB2

|  |  |  |  |  |  |  |  | Lymph nodes |  |
| --- | --- | --- | --- | --- | --- | --- | --- | --- | --- |
| Tumor | Patient | Age | Grade | Stage | Size | pT | pN | Total | positive |
| Ductal | 1 | 66 | 2 | 2 | 3.0 | 2 | X | -- | -- |
|  | 2 | 77 | 2 | 2 | 1.0 | 1 | 1 | 24 | 1 |
|  | 3 | 53 | 2 | 2 | 2.0 | 1 | 1 | 13 | 4 |
|  | 4 | 57 | 2 | 2 | 2.2 | 2 | X | -- | -- |
|  | 5 | 68 | 2 | 1 | 1.1 | 1 | X | -- | -- |
|  | 6 | -- | 2 | 2 | 3.0 | 2 | X | -- | -- |
|  | 7 | 43 | 3 | 3 | 4.5 | 3 | 1 | 14 | 3 |
|  | 8 | 78 | 1 | 2 | 2.2 | 2 | X | -- | -- |
|  | 9 | 57 | 3 | 2 | 1.0 | 1 | 1 | 30 | 3 |
|  | 10 | 60 | 2 | 2 | 4.5 | 2 | 1 | 9 | 3 |
|  | 11 | 54 | 1 | 1 | 1.8 | 1 | X | -- | -- |
|  | 12 | 65 | 1 | 3 | 8.5 | 4 | X | -- | -- |
|  | 13 | 87 | 2 | 2 | 2.3 | 2 | X | -- | -- |
|  | 14 | 79 | 2 | 2 | 3.1 | 2 | X | -- | -- |
|  | 15 | 62 | 1 | 2 | 1.5 | 1 | 1 | 21 | 2 |
|  | 16 | 69 | 1 | 1 | -- | 1 | 0 | 14 | 0 |
|  | 17 | 44 | 3 | 1 | 2.0 | 1 | X | -- | -- |
|  | 18 | 81 | 2 | 2 | 3.0 | 2 | 0 | 2 | 0 |
|  | 19 | 46 | 2 | 1 | 1.5 | 1 | X | -- | -- |
|  | 20 | 47 | 1 | 4 | -- | 4 | 1 | 7 | 1 |
|  | 21 | 88 | 3 | 1 | 0.7 | 1 | X | -- | -- |
|  | 22 | 49 | 2 | 2 | 2.3 | 2 | 1 | 18 | 6 |
|  | 23 | 77 | 2 | 3 | 3.0 | 4 | X | -- | -- |
|  | 24 | 80 | 1 | 1 | 2.0 | 1 | 0 | 1 | 0 |
|  | 25 | 39 | 3 | 2 | 1.5 | 1 | 1 | 19 | 5 |
|  | 26 | 33 | 3 | 2 | 3.0 | 2 | X | -- | -- |
|  | 27 | 71 | 2 | 1 | 0.8 | 1 | 0 | 8 | 0 |
|  | 28 | 61 | 2 | 1 | 2.0 | 1 | 0 | 10 | 0 |
|  | 29 | 84 | 3 | 2 | 2.0 | 2 | X | -- | -- |
|  | 30 | 72 | 2 | 2 | 3.0 | 2 | X | -- | -- |
|  | 31 | 48 | 2 | 2 | 3.5 | 2 | 0 | 19 | 0 |
|  | 32 | 53 | 3 | 2 | 4.0 | 2 | 1 | 14 | 3 |
|  | 33 | 92 | 2 | 2 | 4.0 | 2 | X | -- | -- |
|  | 34 | 62 | 2 | 2 | 2.5 | 2 | X | -- | -- |
|  | 35 | 42 | 2 | 2 | 4.0 | 1 | 1 | 17 | 10 |
|  | 36 | 70 | 3 | 2 | 2.7 | 2 | 0 | 12 | 0 |
|  | 37 | 69 | 2 | 3 | 5.5 | 3 | 0 | 3 | 0 |
|  | 38 | 67 | 2 | 2 | 1.5 | 1 | 1 | 9 | 3 |
|  | 39 | 65 | 2 | 2 | 4.5 | 2 | 1 | 20 | 20 |
|  | 40 | 67 | 1 | 1 | 1.0 | 1 | 0 | 20 | 0 |
|  | 41 | 67 | 2 | 1 | 1.5 | 1 | 0 | 23 | 0 |
|  | 42 | 50 | 2 | 1 | 1.5 | 1 | X | -- | -- |
|  | 43 | 80 | 3 | 3 | 6.5 | 4 | X | -- | -- |
|  | 44 | 62 | 2 | 2 | 3.0 | 2 | 0 | 14 | 0 |
|  | 45 | 80 | 2 | 2 | 1.5 | 1 | 1 | 5 | 3 |
|  | 46 | 85 | 2 | 2 | 4.0 | 2 | X | -- | -- |
|  | 47 | 31 | 3 | 2 | 2.5 | 2 | 1 | 20 | 3 |
|  | 48 | 76 | 2 | 1 | 1.0 | 1 | X | -- | -- |
| Lobular | 1 | 64 | 1 | 1 | 2.0 | 2 | X | -- | -- |
|  | 2 | 75 | 2 | 3 | 7.0 | 3 | 1 | -- | -- |
|  | 3 | 68 | 1 | 2 | 2.5 | 2 | 1 | -- | -- |
|  | 4 | 54 | 2 | 2 | 3.0 | 2 | 1 | -- | -- |
|  | 5 | 66 | 2 | 2 | 3.0 | 2 | 1 | -- | -- |
|  | 6* | 78 | 2 | 2 | 2.5 | 2 | X | -- | -- |
| Cribiform | 1 | 69 | 1 | 1 | 0.9 | 1 | X | -- | -- |
|  | 2 | 65 | 1 | 2 | 2.9 | 2 | X | -- | -- |
| Metaplastic | 1 | -- | 3 | 1 | 2.0 | 1 | X | -- | -- |
| Mucinous | 1 | 68 | 1 | 2 | 5.8 | 3 | X | -- | -- |
| Tubular | 1 | 69 | 1 | 2 | 2.3 | 2 | X | -- | -- |
| Medullary | 1 | 61 | 3 | 1 | 1.9 | 1 | X | -- | -- |
| Fibroadenoma  (control) | 1 | 22 | -- | -- | -- | -- | -- | -- | -- |

Tumor size is represented in centimeters (cm). Patient’s age represented in years. pT: staging designation representing size and infiltration of the tumor. pN: lymph node involvement (0: no involvement / 1 lymph nodes involved). pN = X: none analyzed lymph nodes. Total lymph nodes: number of nodes tested. Positive nodes: number of nodes showing cancer metastasis. (*) destroyed tissue.
