# Supplementary material for: Nuclear Expression of KLF6 Tumor Suppressor Factor Is Highly Associated with Overexpression of ERBB2 Oncoprotein in Ductal Breast Carcinomas
Source: PLoS One. 2010 Jan 28;5(1):e8929. doi: 10.1371/journal.pone.0008929 (PMC2812494; doi:10.1371/journal.pone.0008929)
Supplement: Table S3 — Individual patients' immunohistochemical analysis for KLF6, ERBB2 and Estrogen receptors. KLF6 (nuclear and cytoplasmic) and ERBB2 (cytoplasmic membrane) stain intensity are represented as score scale (0.0–4.0). (+)/(−): indicates if nuclear KLF6 and ERBB2 was considered positive or negative, respectively. (−−) not specified parameter. ND: None determined. (0.14 MB DOC) [file pone.0008929.s006.doc]

Table S3. Individual patients’ immunohistochemical analysis for KLF6, ERBB2 and Estrogen Receptors alpha

|  |  |  | KLF6 |  | Estrogen Receptors |  |
| --- | --- | --- | --- | --- | --- | --- |
| Tumor | Patient Number | ERBB2 | nucleus | Cytoplasm | Nuclear Stain | Cell Percentage |
| Ductal | 1 | 1.8 (+) | 3.2 (+) | 3.4 | Positive | 80 |
|  | 2 | 1.1 (+) | 2.1 (+) | 2.8 | Positive | 80 |
|  | 3 | 3.4 (+) | 2.7 (+) | 3.2 | Positive | 70 |
|  | 4 | 1.6 (+) | 2.6 (+) | 3.1 | Positive | 100 |
|  | 5 | 1.8 (+) | 2.6 (+) | 2.8 | Positive | 100 |
|  | 6 | 0.2 (-) | 2.5 (+) | 1.5 | ND | ND |
|  | 7 | 0.0 (-) | 2.4 (+) | 2.9 | Negative | 0 |
|  | 8 | 1.9 (+) | 2.4 (+) | 2.3 | Negative | 0 |
|  | 9 | 1.3 (+) | 2.3 (+) | 3.1 | Positive | 100 |
|  | 10 | 1.4 (+) | 2.2 (+) | 2.3 | Negative | 0 |
|  | 11 | 1.1 (+) | 2.2 (+) | 2.0 | Positive | 80 |
|  | 12 | 1.6 (+) | 2.0 (+) | 3.3 | Negative | 0 |
|  | 13 | 2.2 (+) | 2.0 (+) | 2.9 | Positive | 100 |
|  | 14 | 1.5 (+) | 2.0 (+) | 2.9 | Positive | 100 |
|  | 15 | 3.3 (+) | 1.8 (+) | 2.7 | Positive | 100 |
|  | 16 | 1.3 (+) | 1.8 (+) | 2.6 | Positive | 100 |
|  | 17 | 2.7 (+) | 1.8 (+) | 2.4 | Negative | 0 |
|  | 18 | 1.9 (+) | 1.8 (+) | 1.6 | Positive | 100 |
|  | 19 | 0.0 (-) | 1.7 (+) | 2.1 | Positive | 100 |
|  | 20 | 1.2 (+) | 1.6 (+) | 2.7 | Negative | 0 |
|  | 21 | 1.6 (+) | 1.5 (+) | 3.4 | Positive | 85 |
|  | 22 | 0.9 (-) | 1.5 (+) | 3.1 | Positive | 87 |
|  | 23 | 0.0 (-) | 1.5 (+) | 1.2 | Positive | 80 |
|  | 24 | 2.0 (+) | 1.3 (+) | 2.5 | Positive | 90 |
|  | 25 | 2.5 (+) | 1.2 (+) | 3.0 | Positive | 60 |
|  | 26 | 1.9 (+) | 1.2 (+) | 2.9 | Negative | 0 |
|  | 27 | 0.8 (-) | 1.2 (+) | 2.4 | Positive | 20 |
|  | 28 | 1.0 (-) | 0.9 (+) | 1.2 | Positive | 95 |
|  | 29 | 0.5 (-) | 0.0 (-) | 3.2 | Positive | 100 |
|  | 30 | 0.0 (-) | 0.0 (-) | 3.1 | Negative | 0 |
|  | 31 | 2.1 (+) | 0.0 (-) | 2.7 | Negative | 0 |
|  | 32 | 0.0 (-) | 0.0 (-) | 2.7 | Negative | 0 |
|  | 33 | 1.1 (+) | 0.0 (-) | 2.4 | Negative | 0 |
|  | 34 | 0.9 (-) | 0.0 (-) | 2.4 | Positive | 80 |
|  | 35 | 1.9 (+) | 0.0 (-) | 2.3 | Negative | 1 |
|  | 36 | 0.9 (-) | 0.0 (-) | 2.3 | Negative | 0 |
|  | 37 | 1.3 (+) | 0.0 (-) | 2.1 | Positive | 100 |
|  | 38 | 0.0 (-) | 0.0 (-) | 2.1 | Positive | 100 |
|  | 39 | 0.9 (-) | 0.0 (-) | 1.9 | Negative | 4 |
|  | 40 | 0.0 (-) | 0.0 (-) | 1.9 | Negative | 0 |
|  | 41 | 1.1 (+) | 0.0 (-) | 1.8 | Positive | 50 |
|  | 42 | 0.0 (-) | 0.0 (-) | 1.8 | Positive | 100 |
|  | 43 | 0.0 (-) | 0.0 (-) | 1.7 | Negative | 0 |
|  | 44 | 0.9 (-) | 0.0 (-) | 1.6 | Positive | 100 |
|  | 45 | 0.0 (-) | 0.0 (-) | 1.1 | Negative | 0 |
|  | 46 | 1.8 (+) | 0.0 (-) | 1.0 | Positive | 70 |
|  | 47 | 0.0 (-) | 0.0 (-) | 1.0 | Positive | 65 |
|  | 48 | 0.0 (-) | 0.0 (-) | 0.7 | Positive | 30 |
| Lobular | 1 | 2.5 (+) | 2.9 (+) | 3.0 | Positive | 85 |
|  | 2 | 1.5 (+) | 1.9 (+) | 2.2 | Positive | 100 |
|  | 3 | 1.3 (+) | 1.0 (+) | 1.3 | Positive | 80 |
|  | 4 | 0.8 (-) | 0.0 (-) | 1.2 | Positive | 80 |
|  | 5 | 0.0 (-) | 0.0 (-) | 0.7 | Negative | 0 |
|  | 6* | -- (--) | -- (--) | -- | Negative | 0 |
| Cribiform | 1 | 1.0 (-) | 1.8 (+) | 2.8 | Positive | 100 |
|  | 2 | 0.0 (-) | 0.2 (+) | 0.9 |  |  |
| Metaplastic | 1 | 1.1 (+) | 2.5 (+) | 3.1 | Negative | 0 |
| Mucinous | 1 | 0.0 (-) | 0.0 (-) | 0.8 | Positive | 60 |
| Tubular | 1 | 0.0 (-) | 0.0 (-) | 1.3 | Positive | 100 |
| Medullary | 1 | 2.0 (+) | 0.0 (-) | 3.2 | Negative | 0 |
| Fibroadenoma  (control) | 1 | 0.0 (-) | 0.0 (-) | 0.6 | Negative | 0 |

KLF6 (nuclear and cytoplasmic) and ERBB2 (cytoplasmic membrane) stain intensity are represented as score scale (0.0-4.0).

(+)/(-): indicates if nuclear KLF6 and ERBB2 was consider positive or negative, respectively.

(--) not specified parameter.

ND: None determined.
