# Supplementary material for: Nuclear Expression of KLF6 Tumor Suppressor Factor Is Highly Associated with Overexpression of ERBB2 Oncoprotein in Ductal Breast Carcinomas
Source: PLoS One. 2010 Jan 28;5(1):e8929. doi: 10.1371/journal.pone.0008929 (PMC2812494; doi:10.1371/journal.pone.0008929)
Supplement: Table S4 — Cell Cycle status of MCF-7 transfected with KLF6 specific siRNA. MCF-7 cells were transfected with scrambled (SCR) or KLF6 specific siRNAs (A and B) as indicated (see also [25]). For flow cytometric cell cycle analysis cells were harvested after 72 h, fixed in 70% ethanol, treated with RNase A (125 mg/ml) and propidium iodide (20 ug/ml), analyzed using a cytometer apparatus and evaluated with Cylchred software. The experiment was conducted in triplicates. Statistical analysis was performed using a one-way ANOVA-Bonferroni analysis where p values<0.05 were considered significant. (0.03 MB DOC) [file pone.0008929.s007.doc]

Table S4. Cell Cycle status of MCF-7 transfected with KLF6 specific siRNA

| Cell Cycle Phase | siRNA | (% ± SD) | p Values |  |
| --- | --- | --- | --- | --- |
| G1 | SCR | 59.3 ± 0.6 | SCR vs KLF6-A | < 0.001 |
|  | KLF6-A | 64.0 ± 1.0 | SCR vs KLF6-B | < 0.001 |
|  | KLF6-B | 63.3 ± 0.6 |  |  |
| S | SCR | 32.3 ± 1.2 | SCR vs KLF6-A | < 0.001 |
|  | KLF6-A | 20.0 ± 1.0 | SCR vs KLF6-B | < 0.05 |
|  | KLF6-B | 25.5 ± 2.9 |  |  |
| G2 | SCR | 8.0 ± 1.7 | SCR vs KLF6-A | < 0.01 |
|  | KLF6-A | 16.3 ± 1.5 | SCR vs KLF6-B | > 0.05 |
|  | KLF6-B | 11.0 ± 3.5 |  |  |

MCF-7 cells were transfected with scrambled (SCR) or KLF6 specific siRNAs (A and B) as indicated (see also (25)). For flow cytometric cell cycle analysis cells were harvested after 72 h, fixed in 70% ethanol, treated with RNase A (125 mg/ml) and propidium iodide (20 ug/ml), analyzed using a cytometer apparatus and evaluated with Cylchred software. The experiment was conducted in triplicates. Statistical analysis was performed using a one-way ANOVA-Bonferroni analysis where p values <0.05 were considered significant.
